# The efficacy of acupuncture for depression-associated chronic pain: a systematic review and meta-analysis

**DOI:** 10.3389/fpsyt.2026.1845974

**Published:** 2026-06-01

**Authors:** Hui Zhao, Yuan Gao, Kun Zhang, Cheng Tang, Weidong Shen

**Affiliations:** 1 Department of Acupuncture, Shuguang Hospital Affiliated to Shanghai University of Traditional Chinese Medicine, Shanghai, China; 2 Department of Tuina, Shuguang Hospital Affiliated to Shanghai University of Traditional Chinese Medicine, Shanghai, China

**Keywords:** acupuncture, chronic pain, depression, meta-analysis, safety

## Abstract

**Objective:**

The comorbidity of pain and depression is prevalent, adding difficulty to the treatment of depression. This systematic review with meta-analysis aims to determine the efficacy and safety of acupuncture in treating depression-associated chronic pain (DACP).

**Methods:**

A comprehensive search was conducted across four international databases, namely PubMed, Embase, Web of Science, and the Cochrane Library, along with four regional databases, including Wanfang Data, CNKI, VIP database, and SinoMed, from inception to March 2025. The Cochrane Risk of Bias 2 tool was utilized to assess risk of bias in the included research articles, and the Grading of Recommendations Assessment, Development, and Evaluations system was employed to evaluate the certainty of evidence. Meta-regression analysis was performed to explore the influence of patient age and treatment duration on the study results, and sensitivity analysis was used to verify the stability of the results. The publication bias was evaluated when the number of included studies exceeded ten. All data analyses were completed using Stata15.1.

**Results:**

Ten randomized controlled trials involving 761 participants were included. Acupuncture combined with conventional medications was more effective than medication alone in improving depressive symptoms (standardized mean difference (SMD): -0.72; 95% confidence interval (CI): -0.91 to -0.53; *P* < 0.01) and reducing pain (SMD: -0.85; 95% CI: -1.36 to -0.34; *P* < 0.01). Head-to-head comparisons revealed that acupuncture is similar to medication in improving the Hamilton Depression Rating Scale scores (SMD: -0.05; 95% CI: -0.61 to 0.51; *P* > 0.05) and the Visual Analogue Scale scores (SMD: -0.33; 95% CI: -0.94 to 0.29; *P* > 0.05), suggesting no statistically significant difference between the two treatments. In contrast, acupuncture was associated with a better safety profile (relative risk: 0.40; 95% CI: 0.27 to 0.60). Further subgroup analysis found the advantage of a 4-week acupuncture treatment in improving depressive symptoms, while longer-term treatment tended to be more effective in relieving pain.

**Conclusions:**

Acupuncture appears to have comparable antidepressant and analgesic effects to conventional oral medications. When applied as an adjuvant therapy, acupuncture may enhance the clinical efficacy of monotherapy for DACP. Regarding treatment duration, a 4-week acupuncture intervention may be superior to a longer cycle (> 4 weeks) in alleviating depressive symptoms, while long-term acupuncture treatment may provide greater benefits in analgesia.

**Systematic Review Registration:**

https://www.crd.york.ac.uk/PROSPERO, identifier CRD420251026454.

## Introduction

Depression stands as a profoundly debilitating mental disorder characterized by enduring low mood, anhedonia, and cognitive decline. In severe cases, it can culminate in suicide or self-harm behaviors (1). With the accelerating pace of life, increasing competitive pressure and heavier economic burdens, the incidence of depression has displayed a significant universal upward trend (2). According to the World Health Organization, depression has affected over 350 million people worldwide and caused approximately one million suicide deaths annually, making it one of the major contributors to global disease burden (3, 4). Depression exhibits distinct chronicity and recurrence, with over 50% of patients relapsing within five years of initial onset, and the recurrence risk increases cumulatively with each subsequent episode (5, 6). Individuals suffering from depression during adolescence are almost triple the risk of relapse in adulthood. Studies have indicated that over half of depression patients experience persistent or recurrent chronic pain, which not only reduces their quality of life but also increases the risk of relapse or recurrence (7). These patients demonstrate stronger treatment resistance and poorer long-term prognosis (8), posing more challenges to clinical treatment.

Chronic pain and depression frequently co-occur and share neurobiological bases, encompassing dysfunctional descending pain modulation systems, pain-depression interactions mediated by genetic and epigenetic modifications, neuroinflammatory responses, and central sensitization (9–11). Epidemiological studies (12, 13) demonstrate that individuals with chronic pain exhibit a threefold increased risk of depression relative to the general population, along with elevated risks of attempted and completed suicide. A cross-sectional survey (14) also confirmed that the prevalence of pain was significantly higher among patients with severe depression compared to the healthy control group. Current management of depression-associated chronic pain (DACP) primarily depends on pharmacotherapy or psychological therapies. However, medications carry risks including frequent adverse effects, suboptimal efficacy, addictive potential, and resistance development (15, 16). Despite cognitive behavioral therapy also holding first-line intervention status, it faces significant limitations including extended treatment duration and substantial dropout rates (17, 18). Consequently, exploring alternative therapeutic approaches capable of simultaneously addressing both depressive symptoms and associated pain with minimal side effects represents a critical research priority.

Given these limitations, acupuncture—a traditional Chinese intervention with great economy and minor adverse effects—demonstrates clinically validated analgesic and antidepressant properties (19, 20). However, DACP-specific evidence remains limited and methodologically suboptimal (21, 22). As a result, robust clinical evidence is required to substantiate acupuncture’s therapeutic value for comorbid depression and chronic pain. This systematic review with meta-analysis therefore evaluates the efficacy and safety profiles of acupuncture both as monotherapy and as part of combined interventions, for DACP management.

## Methods

This study followed the Preferred Reporting Items for Systematic Reviews and Meta-Analyses (PRISMA) reporting guidelines (23). The study protocol was prospectively registered on PROSPERO (Registration Number: CRD420251026454).

### Literature search

Two investigators independently searched four international databases (PubMed, Embase, Web of Science, Cochrane Library) and four Chinese databases (Wanfang, VIP Journal Database, CNKI, SinoMed) from their inception through March 2025. The search strategy incorporated both database-specific Medical Subject Headings terms and free-text keywords related to depression, chronic pain, and acupuncture therapies. No language restrictions were applied. Complete search syntax is provided in Appendix 1.

### Inclusion criteria

1. Study design: Randomized controlled trials (RCTs);
2. Population: Patients meeting depression diagnostic criteria per the Chinese Classification of Mental Disorders (Third Edition), International Classification of Diseases, or Diagnostic and Statistical Manual of Mental Disorders (DSM-III through DSM-5 TR editions), with concurrent chronic pain persisting over three months. There is no restriction on age, gender, or disease severity;
3. Interventions: The experimental group received either acupuncture monotherapy or acupuncture combined with adjuvant therapies, while control groups received any non-acupuncture intervention (e.g., sham acupuncture, pharmacotherapy, or exercise therapy).

### Exclusion criteria

1. Duplicate publications;
2. Studies with unavailable full-text or incomplete outcome data.

### Literature selection and data extraction

All retrieved records were imported into NoteExpress (v4.1.0.10121) for management. Following automatic removal of the duplicates, two investigators (H.Z. and Y.G.) independently screened titles and abstracts to exclude irrelevant literature. Potentially eligible studies underwent full-text assessment, with documented reasons for exclusion. Discrepancies regarding study inclusion were resolved through third-researcher (C.T.) adjudication.

Extracted data included: study characteristics, sample size, publication year, participant gender, mean age, diagnostic criteria, intervention duration, adverse event incidence, along with Visual Analogue Scale (VAS) scores and Hamilton Depression Rating Scale (HAMD) scores post-intervention. Corresponding authors were contacted to retrieve missing data when necessary.

### Quality assessment and evaluation for evidence certainty

The Cochrane Risk-of-Bias Tool 2 (RoB 2) (24) was applied to assess methodological quality of included RCTs. Evaluated domains encompassed: randomization process, deviations from intended interventions, incomplete outcome data, outcome measurement and selective reporting. Studies were categorized as “low risk”, “some concerns”, or “high risk” of bias. Evidence certainty for therapeutic outcomes was evaluated using the Grading of Recommendations Assessment, Development and Evaluation (GRADE) system (25), classifying evidence into four levels: high, moderate, low, or very low. Two investigators (H.Z. and K.Z.) independently applied these tools, with discrepancies resolved through third-reviewer consultation (C.T.).

### Data synthesis and analysis

This meta-analysis defined its primary outcome as the difference in HAMD and VAS scores between acupuncture-treated participants and other intervention groups, with adverse events constituting secondary outcomes. Continuous variables were analyzed using standardized mean differences (SMDs) with 95% confidence intervals (CIs), while dichotomous variables were assessed via risk ratios (RRs). Heterogeneity was evaluated using Cochran’s Q test, quantified by *I*² statistics and *P*-values, with significant heterogeneity (*I*²≥ 50% or *P* < 0.10) prompting use of random-effects models; otherwise, fixed-effects models were applied. To investigate heterogeneity sources, subgroup analyses were conducted by treatment modality and duration, supplemented by meta-regression examining age and treatment duration effects. Sensitivity analysis assessed result robustness, and publication bias was evaluated through funnel plots when over ten studies were included (26). All analyses utilized Stata 15.1 (Stata Corporation, College Station, TX, USA).

## Results

### Search process

**Figure 1:**
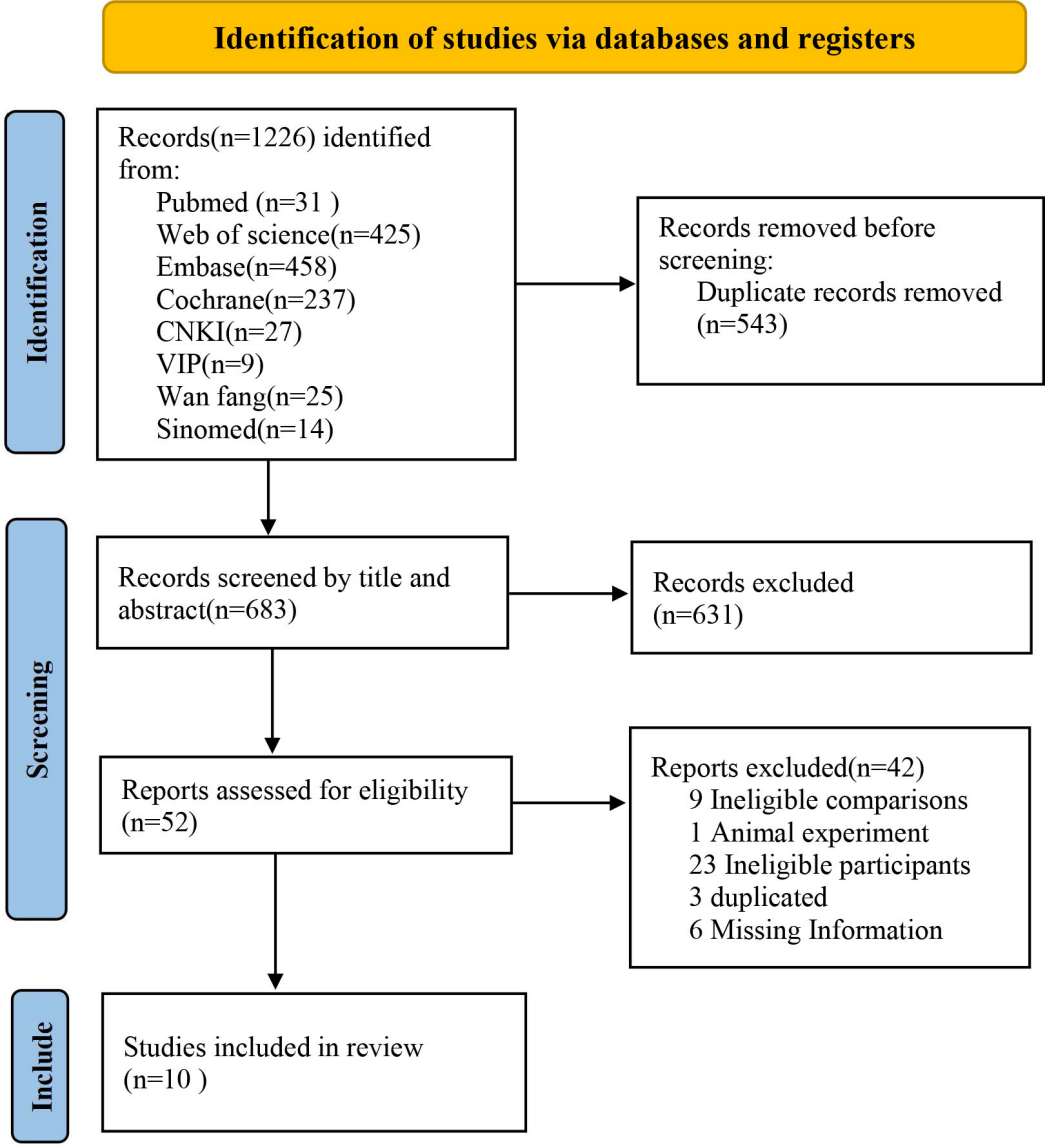
Flow diagram of literature searching and study selection.

A total of 1226 relevant documents were retrieved. Following removal of 543 duplicates, 683 studies underwent title/abstract screening, resulting in exclusion of 631 publications. The remaining 52 full-text articles were comprehensively assessed, ultimately yielding 10 eligible randomized controlled trials (RCTs) encompassing 761 participants for meta-analysis inclusion. The detailed search and filtering process is shown in **Figure 1**.

### Study characteristics

Ten eligible RCTs involving 761 patients with DACP were included. The intervention comparisons comprised: five studies examining acupuncture combined with pharmacotherapy versus medication alone; four comparing acupuncture therapy versus pharmacotherapy; and one evaluating acupuncture with joint mobilization versus isolated joint mobilization. Baihui (GV20), Yintang (EX-HN3), Sishencong (EX-HN1), Neiguan (PC6), Shenmen (HT7), and Taichong (LR3) emerged as the most frequently utilized acupoints. Treatment duration ranged from 4 to 12 weeks. Clinical outcomes were assessed using the HAMD in nine studies and VAS in seven studies to evaluate depressive symptom severity and pain intensity, respectively. Safety assessments in five studies documented adverse events including drowsiness, nausea, vomiting, and dizziness. The main characteristics of all included RCTs are shown in **Table 1**.

**Table 1:** Characteristics of the included studies.

Author (year)	Sample size	Age	Intervention	Comparison	Acupoints	Frequency and duration	Outcome	Adverse events
Huang et al. (2019) (27)	T:55 C:54	T: 45.36 ± 2.78 C: 45.72 ± 2.79	MA+ Deanxit fluoxetine	Deanxit fluoxetine	GV20, EX-HN1, EX-HN3, PC6, HT7	T: 30 min/session, 5 times weekly for 8 weeks. C: Twice a day for 8 weeks.	HAMD VAS	R
Liu et al. (2013) (28)	T:45 C:45	T: 47 ± 8 C: 48 ± 8	MA+SSRIs	SSRIs	DU20, DU29, EX-HN1, DU24, GB20, DU14	T: Every other day for 4 weeks. C: Once daily for 4 weeks.	HAMD VAS SERS	R
Ma et al. (2015) (29)	T: 64 C: 64	T: 39.93 ± 12.93 C: 38.69 ± 14.19	MA+ Duloxetine	Duloxetine	DU29, EX-HN1, PC6, HT7, SP6, LR3	T: 20 min/session, 5 sessions weekly for 8 weeks. C: 40 mg/day initial dose, increase to 60 mg/day within 2 weeks, continued for 8 weeks.	HAMD VAS TESS	R
Zhang et al. (2023) (30)	T: 40 C: 41	T: 59 ± 7 C: 58 ± 7	Warm needle + Sertraline hydrochloride	Sertraline hydrochloride	DU14, DU11, DU9	T: 30 min/session, 5 sessions weekly for 4 weeks. C: 50 mg/day for 4 weeks.	HAMD	NR
Dai et al. (2018) (31)	T:54 C:48	T: 48.7± 12.5 C: 46.3 ± 12.0	MA + Duloxetine or hydrochloride	Duloxetine or hydrochloride	DU20, EX-HN1, PC6, HT7, SP6, LR3	T: 30 min/session, 5 times weekly for 8 weeks. C: 50-250 mg/day for 8 weeks	HAMD VAS	NR
Lee et al. (2023) (32)	T: 23 C: 23	T: 73.78 ± 6.52 C: 74.13 ± 5.63	MA+JM	JM	GB34, ST36, ST40	T: 30 min/session, once weekly for 12 weeks. C: 30 min/session, twice weekly for 12 weeks.	BDI VAS	NR
Zhang et al. (2012) (33)	T: 30 C: 30	NR	MA	Parfluoxetine	DU20, DU16, DU29, EX-HN1, LR3	T: 6-week treatment. C: Once daily for 6 weeks.	HAMD	NR
Yu et al. (2015) (34)	T: 20 C: 20	T: 41 ± 8 C: 40 ± 7	MA	Derisin	DU26, EX-HN1, DU20, HT7, PC6, LR3	T: 50 min/session, 6 times weekly for 8 weeks. C: 1 tablet in the morning and noon for 8 weeks.	HAMD VAS	R
Wei et al. (2019) (35)	T: 35 C: 35	T: 48.23± 9.17 C: 49.18 ± 8.02	EA	Fluoxetine	DU20, DU26, EX-HN1, PC6, LR3, HT7	T: 30 min/session, once daily for 6 weeks. C: 6-week treatment.	HAMD	NR
Cao et al. (2007) (36)	T: 30 C: 30	Range from 20 to 70 years	MA	Derisin	DU20, DU29, EX-HN1, PC6, HT7, LR3	T: 30 min/session, 5 sessions weekly for 4 weeks. C: 2 tablets a day for the first 10 days; afterward, 1 tablet each day, for 4 weeks.	HAMD VAS	R

T, treatment group; C, control group; R, reported; NR, unreported; HAMD, Hamilton Depression Rating Scale; VAS, visual analogue scale; SERS, Rating Scale for Side Effects; MA, manual acupuncture; EA, electroacupuncture; JM, joint movement. SSRIs, Selective Serotonin Reuptake Inhibitors. BDI, Beck Depression Inventory. TESS, Treatment Emergent Symptom Scale.

### Risk-of-bias assessment

**Figure 2:**
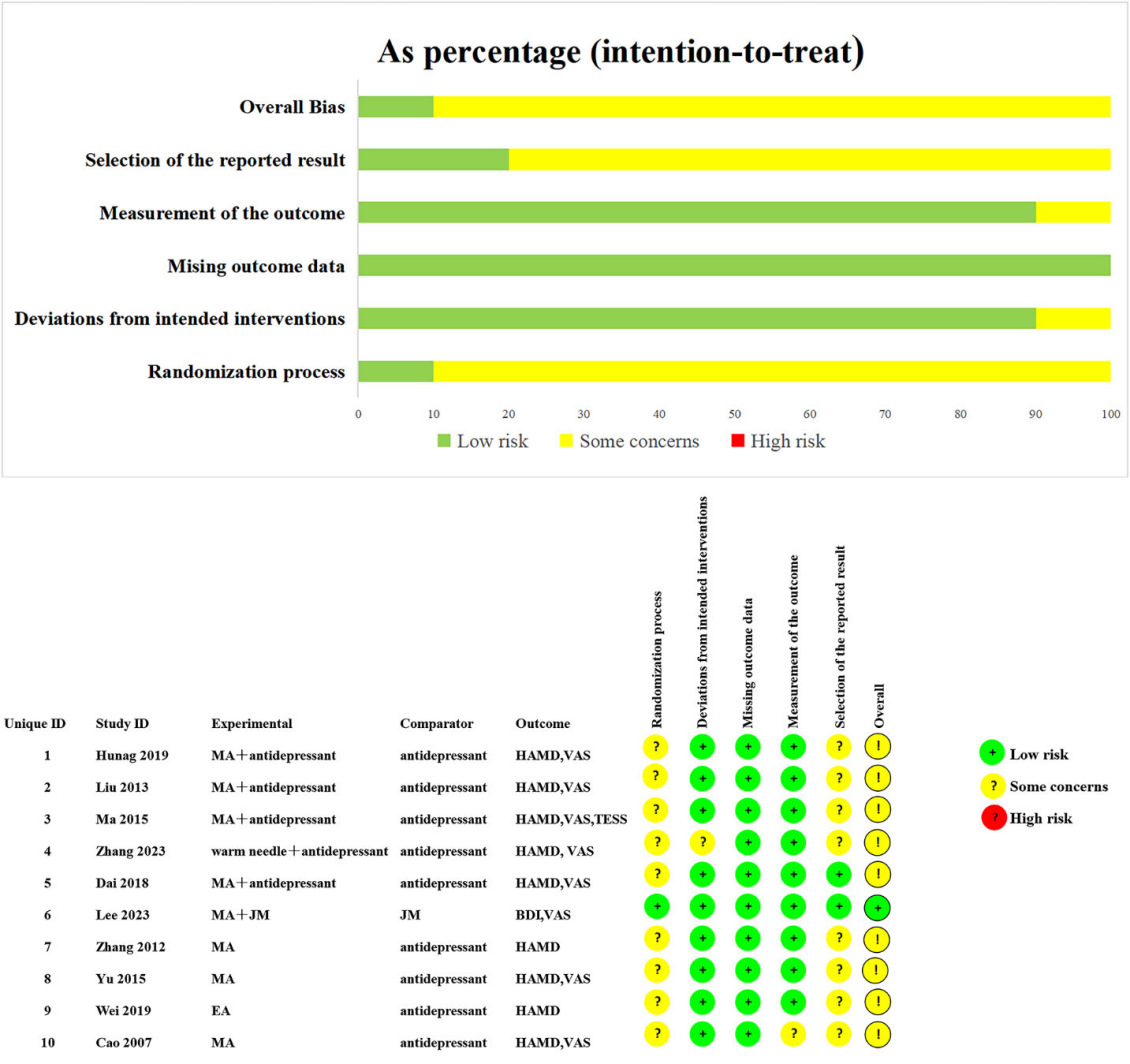
Detailed risk of bias assessment. HAMD, Hamilton depression rating scale; VAS, visual analogue scale; MA, manual acupuncture; EA, electroacupuncture; JM, joint movement. BDI, Beck depression inventory. TESS, treatment emergent symptom scale.

Regarding overall risk of bias, one included study was assessed as low-risk, while the remaining nine raised some concerns by the RoB 2 tool. All studies demonstrated low risk of bias for missing outcome data. However, critical bias risks primarily emerged in randomization process and selection of the reported result. Only one study adequately described allocation concealment methodology while strictly following the registered protocol. Additionally, one trial potentially deviated from intended interventions, and another exhibited possible outcome measurement bias. Detailed ratings are presented in **Figure 2**.

### The antidepressant effect of acupuncture on DACP

**Figure 3:**
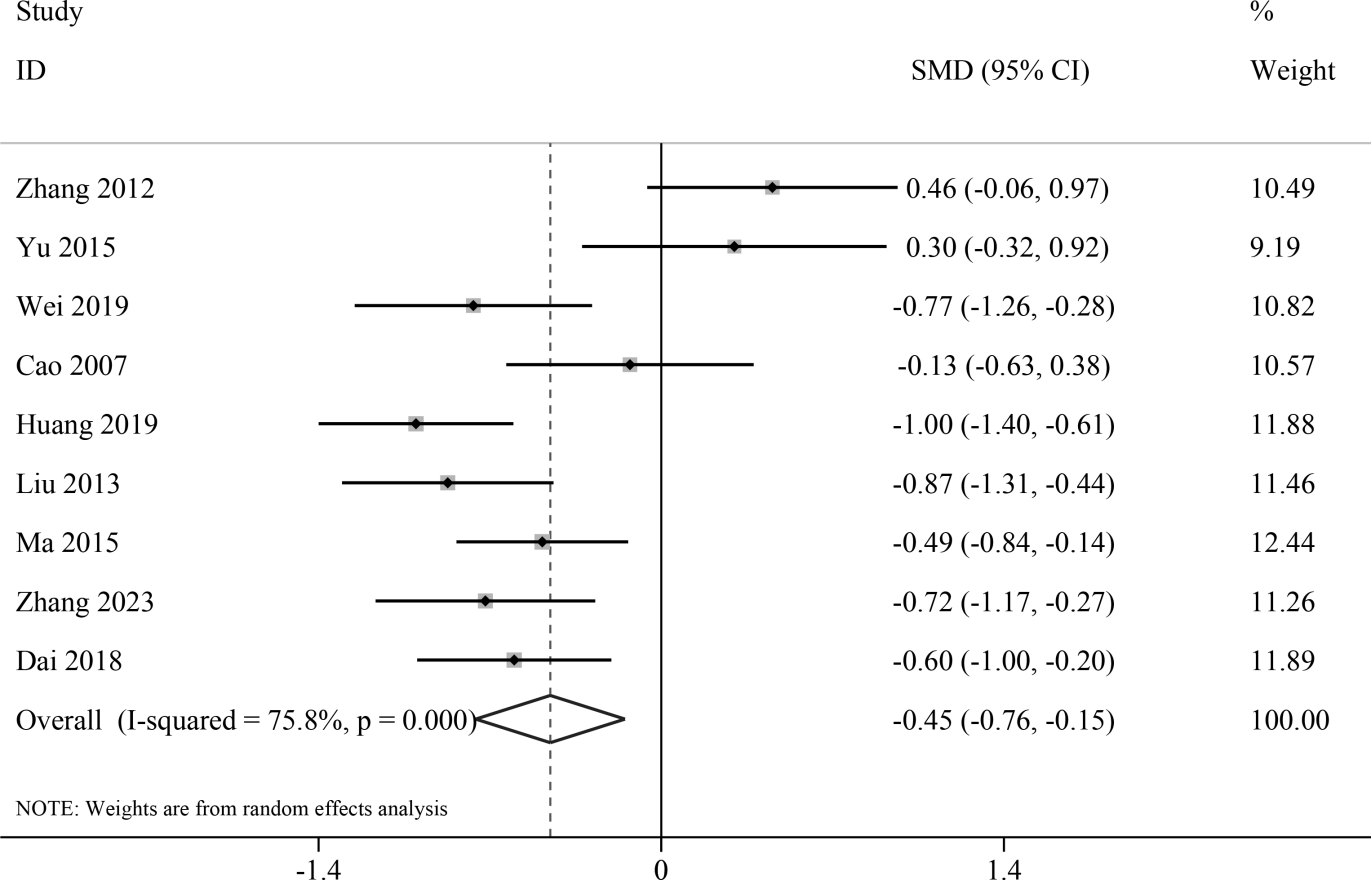
Meta-analysis on the effects of acupuncture on Hamilton depression rating scale (HAMD). SMD, standardized mean difference; CI, confidence interval.

Across the nine studies utilizing the HAMD, acupuncture interventions—whether administered alone or in combination—were associated with greater reductions in depression scores compared to control groups (SMD: -0.45; 95% CI: -0.76 to -0.15; *P* < 0.01). However, substantial heterogeneity was observed among these studies (*I*² = 75.8%, *P* < 0.01) (**Figure 3**).

### Subgroup analysis of HAMD scores

**Figure 4:**
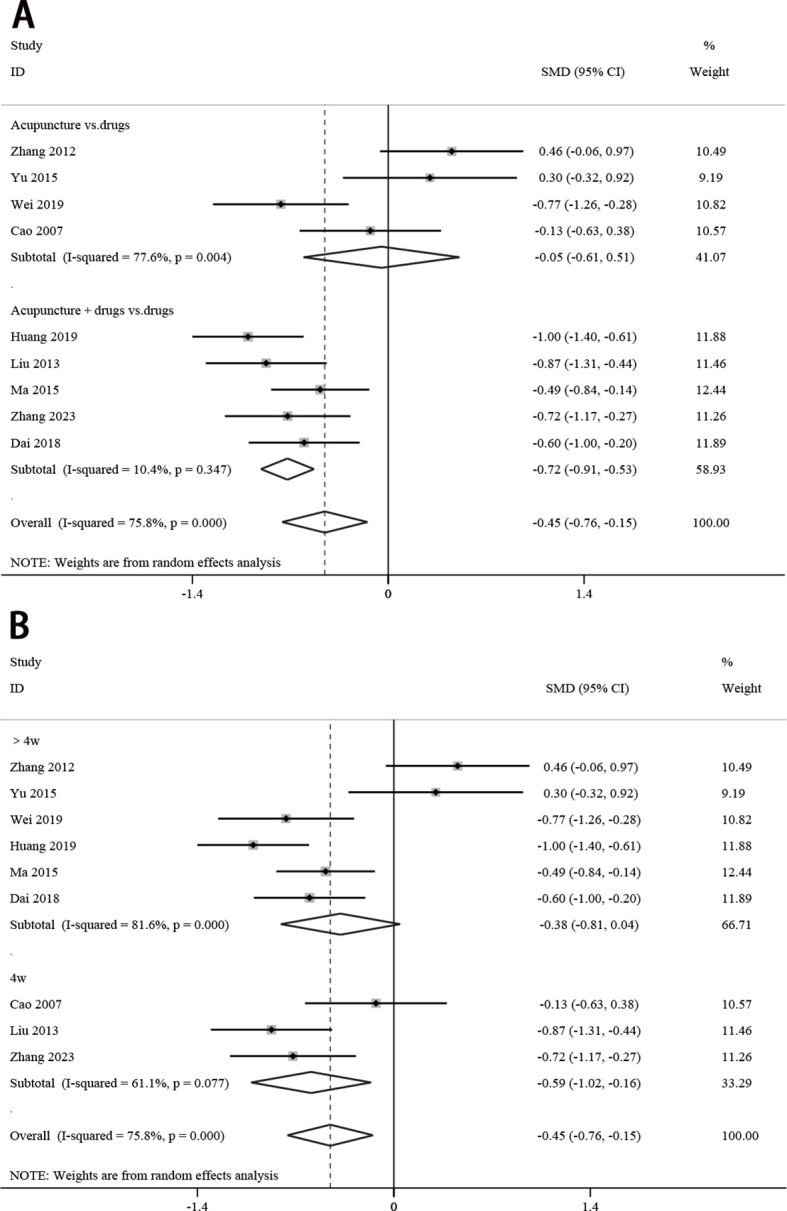
Subgroup analysis for Hamilton depression rating scale (HAMD) based on intervention type **(A)** and intervention duration **(B)**. SMD, standardized mean difference; CI, confidence interval.

Subgroup analysis by intervention type demonstrated that acupuncture combined with pharmacotherapy yielded significantly greater reductions in HAMD scores versus pharmacotherapy alone (SMD: -0.72; 95% CI: -0.91 to -0.53; *P* < 0.01), supporting its adjunctive value in antidepressant therapy for DACP patients. Acupuncture monotherapy demonstrated similar improvements in HAMD compared to conventional medication (SMD: -0.05; 95% CI: -0.61 to 0.51; *P*>0.05, **Figure 4A**).

Subgroup analysis based on treatment duration suggested a trend toward improved HAMD scores with 4-week interventions compared to longer durations (SMD: -0.45; 95% CI: -0.76 to -0.15; P < 0.01), but a formal statistical test for subgroup differences was not conducted (**Figure 4B**).

### The analgesic effect of acupuncture on DACP

**Figure 5:**
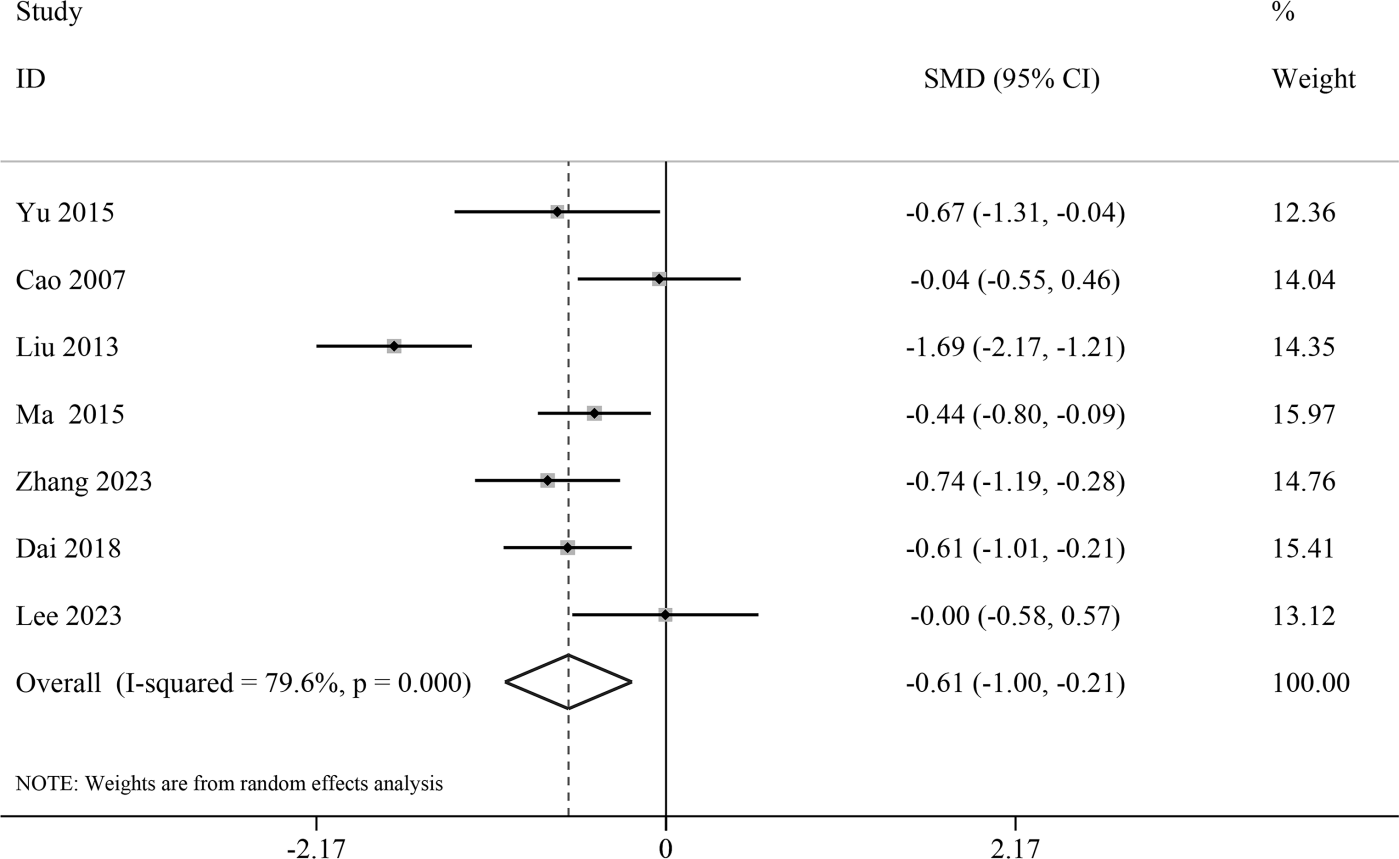
Meta-analysis on the effects of acupuncture on visual analogue scale (VAS). SMD, standardized mean difference; CI, confidence interval.

Among seven studies assessing pain by VAS scores, acupuncture interventions applied as monotherapy or part of combination therapy demonstrated significantly greater pain reduction compared to controls (SMD: -0.61; 95% CI: -1.00 to -0.21). However, substantial heterogeneity was observed among these studies (*I*² = 79.6%, *P* < 0.01; **Figure 5**).

### Subgroup analysis of VAS scores

**Figure 6:**
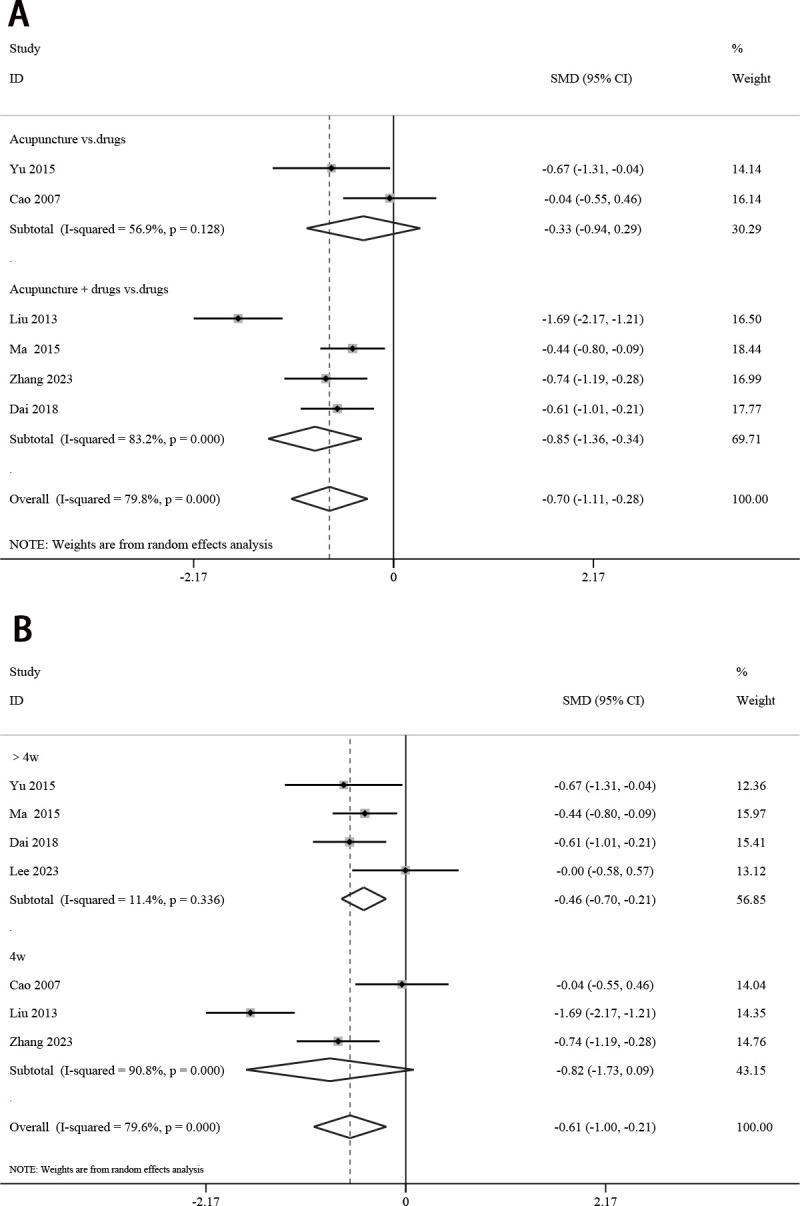
Subgroup analysis for visual analogue scale based on intervention type **(A)** and intervention duration **(B)**. SMD, standardized mean difference; CI, confidence interval.

Subgroup analysis of VAS scores by intervention type revealed greater analgesic effects for acupuncture-pharmacotherapy combination versus pharmacotherapy alone (SMD: -0.85; 95% CI: -1.36 to -0.34; P < 0.01), supporting a potential adjunctive analgesic role of acupuncture in DACP management. When applied as monotherapy, acupuncture demonstrated similar analgesic efficacy to pharmacotherapy (SMD: -0.33; 95% CI: -0.94 to 0.29; P > 0.05; **Figure 6A**).

In subgroup analysis based on treatment duration, acupuncture interventions lasting over four weeks showed statistically significant improvements in VAS scores (SMD: -0.46; 95% CI: -0.70 to -0.21; *P* < 0.05), whereas outcomes of four-week interventions showed debated analgesic effects (SMD: -0.82; 95% CI: -1.73 to 0.09; *P* > 0.05; **Figure 6B**).

### Adverse reactions

**Figure 7:**
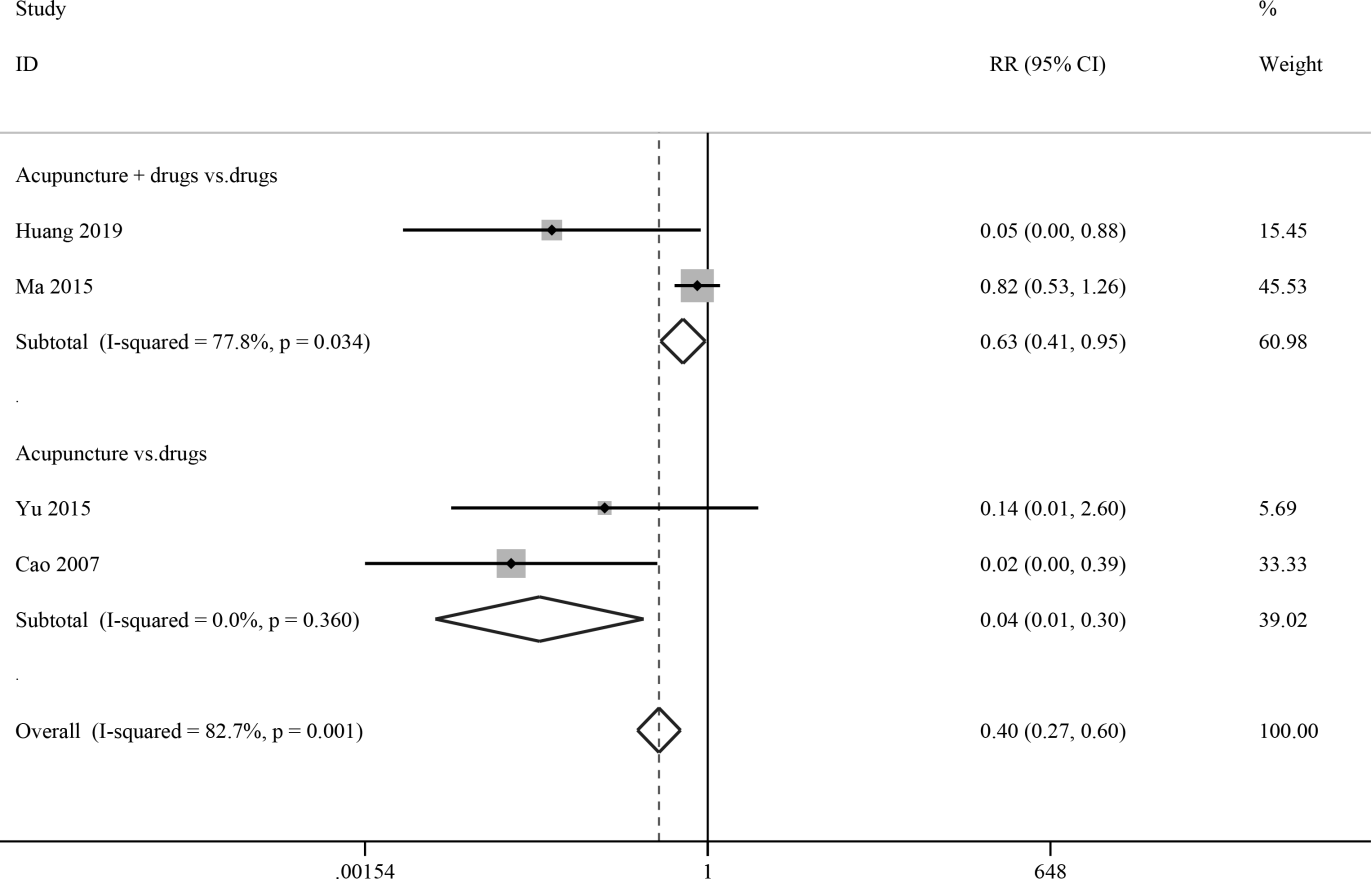
Meta-analysis on the adverse effects with subgroup analysis based on intervention types. RR, risk ratio; CI, confidence interval.

Five studies documented adverse events, with one employing the Asberg Side Effects Rating Scale and another utilizing the Treatment Emergent Symptom Scale for standardized assessment. Four studies quantified adverse reaction incidence, revealing dizziness, nausea and vomiting as the most prevalent post-treatment effects. Subgroup analysis demonstrated significantly lower adverse reaction incidence with acupuncture applied as monotherapy or adjunctive treatment, compared to control groups (RR: 0.40; 95% CI: 0.27 to 0.60; *P* < 0.01; **Figure 7**).

### Meta-regression

Meta-regression analysis was adopted to assess the influence of patient age and treatment duration on therapeutic outcomes. The results demonstrated that age significantly affected the evaluation of HAMD (*P* < 0.05), while treatment duration showed no substantial association. For VAS outcomes, neither age nor treatment duration significantly influenced the outcome evaluations (*P* > 0.05; **Table 2**).

**Table 2:** Meta-regression of HAMD and VAS scores.

Outcome	ES	exp (b)	Std. Err.	t	*P*>|t|	95% Confidence interval	
						Lower limit	Upper limit
HAMD	Age	-0.3593001	0.0908232	-3.96	0.005	-0.574063	0. 1445373
	Course	0.1050369	0.2174961	0.48	0.646	-0.427157	0.6372308
	cons	-0.0757033	0.4210326	-0.18	0.863	-1.105933	0.9545263
VAS	Age	-0.1130547	0.2283035	-0.50	0.646	-0.7469268	0.5208173
	Course	0.3945994	0.4717969	0.84	0.450	-0.9153187	1.704518
	cons	-1.029413	0.8607582	-1.20	0.298	-3.419261	1.360435

HAMD, Hamilton Depression Rating Scale; VAS, visual analogue scale; Std. Err., standard error; ES, effect size. Exp, exponential function.

### Sensitivity analyses

**Figure 8:**
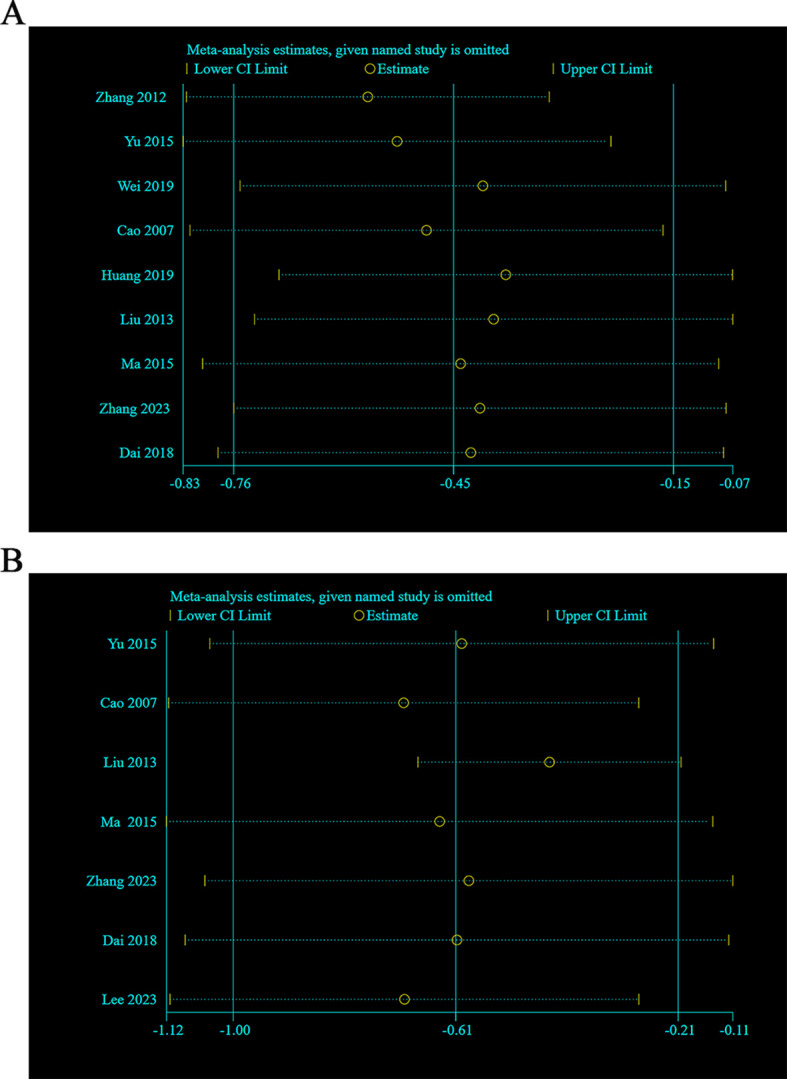
Sensitivity analysis for Hamilton Depression Scale **(A)** and visual analogue scale **(B)** by the leave-one-out method. CI, confidence interval.

Sensitivity analysis was conducted using the leave-one-out method in the evaluation of HAMD and VAS. The stability of the present results was confirmed as effect estimates remained robust following sequential exclusion of individual studies (**Figure 8**).

### Evidence certainty

The certainty of evidence for both primary outcomes was assessed as low to very low (**Table 3**). Downgrading decisions were attributed to RoB, inconsistency, and imprecision. Publication bias did not contribute to downgrading given the limited number of studies. Indirectness was not considered a downgrading factor due to this review’s strict adherence to pre-specified population, intervention, comparator, and outcome criteria. The detailed evaluation process of GRADE is shown in Appendix 2.

**Table 3:** GRADE summary of HAMD and VAS for all comparisons among trials included.

Outcome	Comparisons	Studies	Effect estimates (95% CI)	*I*^2^ (%)	Quality of evidence
HAMD	Acupuncture + drugs versus drugs	Huang et al., 2019 (27)	-0.45 (-0.76, -0.15)	10.4	⊕OOO Low
		Liu et al., 2013 (28)			
		Ma et al., 2015 (29)			
		Zhang et al., 2023 (30)			
		Dai et al., 2018 (31)			
	Acupuncture versus drugs	Zhang et al., 2012 (33)	-0.05 (-0.61, 0.51)	77.6	⊕⊕OO Very Low
		Yu et al., 2015 (34)			
		Wei et al., 2019 (35)			
		Cao et al., 2007 (36)			
VAS	Acupuncture + drugs versus drugs	Liu et al., 2013 (28)	-0.85 (-1.36, -0.34)	83.2	⊕OOO Very Low
		Ma et al., 2015 (29)			
		Zhang et al., 2023 (30)			
		Dai et al., 2018 (31)			
	Acupuncture versus drugs	Yu et al., 2015 (34)	-0.33 (-0.94, 0.25)	56.9	⊕⊕OO Low
		Cao et al., 2007 (36)			

HAMD, Hamilton depression rating scale; VAS, visual analogue scale; CI, confidence intervals.

### Publication bias

Due to the limited number of studies (n = 9) evaluating HAMD scores, formal statistical assessments of publication bias such as funnel plot analysis or Egger’s test were not performed, as these methods are unreliable with fewer than 10 studies.

## Discussion

Depression and chronic pain represent major global health burdens with significant comorbid characteristics. Existing evidence indicates that multidimensional pain intensity and frequency constitute the strongest predictors of late-life depression (37, 38). Mediated through complex bidirectional neurobiological interactions, these interrelationships between depression and chronic pain correlate with multisystem pathophysiology, more severe clinical manifestations, and poorer prognostic outcomes (39, 40). Although multiple RCTs confirm acupuncture’s established efficacy in alleviating depressive symptoms and pain (41–43), a scarcity of high-quality systematic evaluations persists regarding its comprehensive therapeutic effects on DACP. Rigorous investigations examining acupuncture’s efficacy and safety profiles for DACP management remain imperative.

The included studies comprised both add-on trials (acupuncture plus pharmacotherapy vs. pharmacotherapy alone) and head-to-head trials (acupuncture monotherapy vs. pharmacotherapy), which address distinct clinical questions. Subgroup analyses by intervention type were conducted to account for these differences. Add-on trials showed that acupuncture combined with pharmacotherapy significantly reduced HAMD and VAS scores compared to pharmacotherapy alone, supporting its adjunctive benefit. Head-to-head trials demonstrated comparable efficacy between acupuncture monotherapy and pharmacotherapy. This distinction allows accurate interpretation of incremental and relative effects and justifies their combined synthesis, ensuring methodological rigor and clinical relevance. We acknowledge the potential impact of this design diversity on the conclusions and carefully considered it when synthesizing the results. Diverging from prior meta-analyses (21, 22) primarily examining pain-related depression, this investigation constitutes the first quantitative synthesis targeting the efficacy of acupuncture on DACP specifically. Acupuncture was associated with improvements in both pain and depressive symptoms, although the certainty of evidence ranged from low to very low. These findings align with established evidence, providing preliminary evidence supporting its potential clinical application in DACP management.

Depression and chronic pain share complex neuropathological mechanisms. Research (44) demonstrates that persistent negative emotions compromise endogenous analgesia systems by lowering pain thresholds, triggering chronic pain development. Conversely, chronic pain promotes depression through suppressed dopaminergic neuron activity in the midbrain ventral tegmental area, a key region regulating nociception and emotional processing (45). Neuroinflammation mediated by glial cell activation constitutes a vital neuropathological feature in depression. The released inflammatory mediators, such as tumor necrosis factor-α, interleukin-1β, interleukin-6 and interferon-γ, concurrently damage limbic emotional circuits and periaqueductal gray-spinal descending analgesic pathways (46, 47). Moreover, central sensitization driven by neuroinflammation amplifies nociception, thereby exacerbating chronic pain. Emerging neuroanatomical evidence (48, 49) reveals a substantial overlap in brain parenchymal structures involved in negative emotions and chronic pain, specifically involving brain regions such as the hippocampus, amygdala, anterior cingulate cortex, and dorsomedial prefrontal cortex (dmPFC) (50, 51). Furthermore, some studies (52) have also emphasized the role of thalamus in depression-pain comorbidities through central regulatory mechanisms. Concerning the neurotransmitter, chronic pain impairs amygdala serotonergic (5-HT) transmission, attenuating negative emotion inhibition while disrupting midbrain-limbic dopamine pathways. Resultant dopamine/5-HT dysregulation compromises dorsolateral PFC-mediated nociceptive processing and emotional regulation (53), establishing a self-perpetuating pain-depression cycle that heightens treatment resistance in DACP. However, current literature predominantly addresses neuropathic pain-related depression, while DACP-specific clinical investigations remain scarce.

The present meta-analysis suggests that acupuncture may enhance clinical outcomes as an adjunctive therapy while exhibiting comparable antidepressant and analgesic efficacy to conventional pharmacotherapy. As a crucial component of traditional Chinese medicine, acupuncture achieves neural-immune modulation through acupoint stimulation, offering rapid onset, target specificity, and favorable safety profiles that contribute to its clinical adoption (54). Notably, acupuncture demonstrated substantially fewer adverse events compared to pharmacotherapy, with no serious adverse events reported in the trials analyzed, which somewhat reinforces its clinical safety profile.

While acupuncture’s mechanisms in DACP remain incompletely elucidated, substantial evidence (55–57) confirms its ability to remodel aberrant neural circuitry, including infralimbic amygdala-basolateral amygdala connectivity, prefrontal monoaminergic pathways, and central amygdala neuronal activity, to exert therapeutic effects. Acupuncture further achieves antidepressant and analgesic effects by normalizing hypothalamic-pituitary-adrenal axis feedback to inhibit glucocorticoid receptor-mediated cortisol neurotoxicity while concurrently activating endogenous opioid systems (58). Additionally, acupuncture therapy can dynamically balance noradrenergic ascending projections with serotonergic descending inhibition, while restoring hippocampal glutamatergic/GABAergic synaptic homeostasis (59). At the molecular level, acupuncture also activates brain-derived neurotrophic factor/tropomyosin-related kinase B/cAMP-response element binding protein (BDNF/TrkB/CREB) signaling cascades and phosphoinositide 3-kinase/protein kinase B PI3K/Akt pathways (60, 61), collectively establishing the neurobiological foundation for its antidepressant-analgesic efficacy.

Current evidence lacks consensus regarding the temporal characteristics of acupuncture’s analgesic versus antidepressant effects. Our findings suggest acupuncture may yield superior long-term analgesia coupled with short-term antidepressant efficacy. This temporal divergence likely stems from distinct mechanisms: sustained analgesia involves synaptic plasticity modulation and prolonged glial cell regulation (62), whereas immediate antidepressant effects rely predominantly on rapid activation of the monoaminergic system and the BDNF-TrkB-CREB pathway (63). In addition, gene expression and proteomics may also be influencing factors of the time-response characteristics of acupuncture treatment (64).

This study has several limitations that should be carefully considered when interpreting the findings. Firstly, the conclusions drawn should be regarded as preliminary due to the limited availability of high-quality clinical evidence. In addition, the certainty of evidence for several outcomes was rated as low or very low, which may limit the robustness and reliability of the conclusions. Therefore, these findings should be interpreted with caution. Validation through larger sample sizes and more rigorously designed RCTs is essential for confirming these findings. Secondly, substantial heterogeneity was observed across the included trials, potentially arising from variations in patient characteristics, intervention protocols, and methodological quality. Although subgroup analyses and meta-regression were performed, the underlying sources of heterogeneity could not be fully explained.

## Conclusion

As an adjunctive therapy, acupuncture appears to be associated with greater improvements compared with first-line therapies alone, particularly suggesting potential long-term analgesic benefits and short-term antidepressant effects. When administered as monotherapy, acupuncture may provide similar improvements compared with conventional antidepressant medications. While acupuncture’s efficacy for Depression-associated Chronic Pain (DACP) remains somewhat constrained and continues to invite scholarly debate, it is suggested to maintain a favorable safety profile with minimal adverse effects. To further evaluate and potentially integrate acupuncture into DACP patient management, systematic randomized controlled trials and large-scale clinical datasets are needed.

## Data Availability

The original contributions presented in the study are included in the article/**Supplementary Material**. Further inquiries can be directed to the corresponding author.
