# Supplementary material for: The efficacy of acupuncture for depression-associated chronic pain: a systematic review and meta-analysis
Source: Front Psychiatry. 2026 Jun 1;17:1845974. doi: 10.3389/fpsyt.2026.1845974 (PMC13265526; doi:10.3389/fpsyt.2026.1845974)
Supplement: Supplementary file 1 [file SupplementaryFile1.docx]

**Appendix 1 Search terms and strategies of pubmed**

**Pubmed**

#1 "Depressive Disorder"[MeSH Terms] OR "depressive disorder, major"[MeSH Terms] OR "Depression"[MeSH Terms]

#2 ("Depressive Disorder"[MeSH Terms] OR "depressive disorder, major"[MeSH Terms] OR "Depression"[MeSH Terms] OR "depressive symptom*"[Title/Abstract] OR "symptom depressive"[Title/Abstract] OR "emotional depression"[Title/Abstract] OR "depression emotional"[Title/Abstract] OR "depressive disorder*"[Title/Abstract] OR "disorder* depressive"[Title/Abstract] OR "neurosis depressive"[Title/Abstract] OR "depressive neuroses"[Title/Abstract] OR "depressive neurosis"[Title/Abstract] OR "neuroses depressive"[Title/Abstract] OR "depression* endogenous"[Title/Abstract] OR "endogenous depression*"[Title/Abstract] OR "melancholia*"[Title/Abstract] OR "unipolar depression*"[Title/Abstract] OR "depression* unipolar"[Title/Abstract] OR "depressive syndrome*"[Title/Abstract] OR "syndrome* depressive"[Title/Abstract] OR "clinical depression"[Title/Abstract] OR "depression involutional"[Title/Abstract] OR "involutional psychoses"[Title/Abstract] OR "psychoses involutional"[Title/Abstract])

#3 #1 AND #2

#4 "Chronic Pain"[MeSH Terms]

#5 ("Chronic Pain"[MeSH Terms] OR "pain chronic"[Title/Abstract] OR "widespread chronic pain"[Title/Abstract] OR "widespread chronic"[Title/Abstract] OR "chronic primary pain"[Title/Abstract] OR "pain chronic primary"[Title/Abstract] OR "primary pain chronic"[Title/Abstract] OR "chronic secondary pain"[Title/Abstract] OR "secondary pain chronic"[Title/Abstract])

#6 #4 AND #5

#7 "Acupuncture"[MeSH Terms] OR "Acupuncture Therapy"[MeSH Terms]

#8 ("Acupuncture"[MeSH Terms] OR "Acupuncture Therapy"[MeSH Terms] OR "Acupuncture"[Title/Abstract] OR "Electroacupuncture"[Title/Abstract] OR "scalp needle"[Title/Abstract] OR "Pharmacopuncture"[Title/Abstract] OR "pharmacoacupuncture treatment"[Title/Abstract] OR "pharmacoacupuncture therapy"[Title/Abstract] OR "Acupotomy"[Title/Abstract] OR "Acupotomies"[Title/Abstract])

#9 #7 AND #8

#10 #3 AND #6 AND #9

**Appendix 2 The GRADE approach to evidence synthesis and operationalization of criteria items**

| **acupuncture＋drugs compared to drugs for DRCP** | | | | | | |
| --- | --- | --- | --- | --- | --- | --- |
| **Patient or population:** patients with DRCP **Settings:**  **Intervention:** acupuncture＋drugs **Comparison:** drugs | | | | | | |
| **Outcomes** | **Illustrative comparative risks* (95% CI)** | | **Relative effect (95% CI)** | **No of Participants (studies)** | **Quality of the evidence (GRADE)** | **Comments** |
|  | Assumed risk | Corresponding risk |  |  |  |  |
|  | **Drugs** | **Acupuncture＋drugs** |  |  |  |  |
| **HAMD** | The mean hamd in the control groups was **0** | The mean hamd in the intervention groups was **0.72 standard deviations lower** (0.91 to 0.34 lower) |  | 511 (5 studies) | ⊕⊕⊝⊝ **low**^1,2,3^ | 1,2,3 |
| **VAS** |  | The mean vas in the intervention groups was **0.85 standard deviations lower** (1.36 to 0.34 lower) |  | 401 (4 studies) | ⊕⊝⊝⊝ **very low**^1,4,5^ | 4,5 |
| *The basis for the **assumed risk** (e.g. the median control group risk across studies) is provided in footnotes. The **corresponding risk** (and its 95% confidence interval) is based on the assumed risk in the comparison group and the **relative effect** of the intervention (and its 95% CI). **CI:** Confidence interval; | | | | | | |
| **GRADE Working Group grades of evidence** **High quality:** Further research is very unlikely to change our confidence in the estimate of effect.  **Moderate quality:** Further research is likely to have an important impact on our confidence in the estimate of effect and may change the estimate. **Low quality:** Further research is very likely to have an important impact on our confidence in the estimate of effect and is likely to change the estimate.  **Very low quality:** We are very uncertain about the estimate. | | | | | | |
| **Footnotes (reasons for downgrading the evidence):**  ^1^ No explanation provided in the original study – The study did not provide clear justification or detail for the reported results. ^2^ Small sample size – The total number of participants in the study is relatively small, which may reduce the precision and reliability of the effect estimate.  ^3^ Large observed effect – The magnitude of the observed effect is unusually large, which may indicate potential overestimation or bias.  ^4^ Unclear allocation concealment – The method of randomization or allocation concealment was not reported, which may increase risk of selection bias. ^5^ High heterogeneity (I²> 75%)–Substantial variation in effect estimates among included studies reduces confidence in the pooled result. | | | | | | |

| **Acupuncture compared to drugs for DRCP** | | | | | | |
| --- | --- | --- | --- | --- | --- | --- |
| **Patient or population:** patients with DRCP **Settings:**  **Intervention:** Acupuncture **Comparison:** drugs | | | | | | |
| **Outcomes** | **Illustrative comparative risks* (95% CI)** | | **Relative effect (95% CI)** | **No of Participants (studies)** | **Quality of the evidence (GRADE)** | **Comments** |
|  | Assumed risk | Corresponding risk |  |  |  |  |
|  | **Drugs** | **Acupuncture** |  |  |  |  |
| **HAMD** |  | The mean hamd in the intervention groups was **0.05 standard deviations lower** (0.61 lower to 0.51 higher) |  | 230 (4 studies) | ⊕⊝⊝⊝ **very low**^1,2^ | 1,2 |
| **VAS** |  | The mean vas in the intervention groups was **0.33 standard deviations lower** (0.94 lower to 0.29 higher) |  | 100 (2 studies) | ⊕⊕⊝⊝ **low**^1,3^ | 1,3 |
| *The basis for the **assumed risk** (e.g. the median control group risk across studies) is provided in footnotes. The **corresponding risk** (and its 95% confidence interval) is based on the assumed risk in the comparison group and the **relative effect** of the intervention (and its 95% CI).  **CI:** Confidence interval; | | | | | | |
| GRADE Working Group grades of evidence **High quality:** Further research is very unlikely to change our confidence in the estimate of effect.  **Moderate quality:** Further research is likely to have an important impact on our confidence in the estimate of effect and may change the estimate. **Low quality:** Further research is very likely to have an important impact on our confidence in the estimate of effect and is likely to change the estimate. **Very low quality:** We are very uncertain about the estimate. | | | | | | |
| **Footnotes (reasons for downgrading the evidence):**  ^1^ Allocation concealment unclear: The studies did not provide sufficient information on how the randomization sequence was concealed, which may increase the risk of selection bias.  ^2^ High heterogeneity (I² > 75%): Considerable variation in effect estimates among included studies reduces the confidence in the pooled result. ^3^ Moderate heterogeneity (I² > 50%): Moderate variation in effect estimates may slightly reduce the confidence in the pooled effect estimate. | | | | | | |
